# Plasma Protein Profiles Differ Between Women Diagnosed with Cervical Intraepithelial Neoplasia (CIN) 1 and 3

**Published:** 2007-02-27

**Authors:** Chandrika J. Piyathilake, Denise K. Oelschlager, Sreelatha Meleth, Edward E. Partridge, William E. Grizzle

**Affiliations:** 1 Department of Nutrition Sciences; 2 Department of Pathology; 3 Department of Biostatistics; 4 Department of Obstetrics & Gynecology, University of Alabama at Birmingham, Birmingham, Alabama 35294

**Keywords:** cervical, neoplasia, protein profiles, SELDI

## Abstract

Early detection of precancerous cells in the cervix and their clinical management is the main purpose of cervical cancer prevention and treatment programs. Cytological findings or testing for high risk (HR)-human papillomavirus (HPV) are inadequately sensitive for use in triage of women at high risk for cervical cancer. The current study is an exploratory study to identify candidate surface-enhanced laser desorption/ionization (SELDI) time of flight (TOF) mass spectrometry (MS) protein profiles in plasma that may distinguish cervical intraepithelial neoplasia (CIN 3) from CIN 1 among women infected with HR-HPV. We evaluated the SELDI-TOF-MS plasma protein profiles of HR-HPV positive 32 women with CIN 3 (cases) and 28 women with CIN1 (controls). Case-control status was kept blinded and triplicates of each sample and quality control plasma samples were randomized and after robotic sample preparations were run on WCX2 chips. After alignment of mass/charge (m-z values), an iterative method was used to develop a classifier on a training data set that had 28 cases and 22 controls. The classifier developed was used to classify the subjects in a test data set that has six cases and six controls. The classifier separated the cases from controls in the test set with 100% sensitivity and 100% specificity suggesting the possibility of using plasma SELDI protein profiles to identify women who are likely to have CIN 3 lesions.

## Introduction

Pap smear is the most commonly used diagnostic test used to identify women who are at risk of developing cervical cancer. An estimated 50 million Pap smears are performed each year in the United States (Walsh, 1998) and 6% of these are diagnosed as low-grade squamous intraepithelial lesions (LSILs) or as atypical squamous cells of undetermined significance (ASCUS) (Paavonen et al. 1990). Although there is general consensus that cytologically diagnosed high-grade squamous intraepithelial lesions (HSILs) should be evaluated by colposcopy and biopsy, there has been no consensus as to the clinical management of women diagnosed with ASCUS or LSILs. Current options include immediate colposcopy and directed biopsy or follow-up with repeat cytology every 4–6 months and colposcopy with repeated abnormal cytology. The annual cost associated with these treatment options in the U.S.A. is estimated to be over one billion dollars (Schechter, 1996). Since HR-HPV is the main risk factor for cervical cancer, recent studies have investigated the use of HPV testing in the clinical management of these lesions. The ASCUS/LSIL Triage Study for cervical cancer (ALTS) conducted by the NCI demonstrated that HPV testing is sensitive in detecting underlying precancerous lesions (CIN 2 or 3) among women with a Pap test diagnosis of ASCUS (Solomon et al. 2001), but HPV testing is not useful for women with a Pap test diagnosis of LSIL (ALTS investigators, 2000). Therefore, novel markers with higher specificity for the presence of precancerous lesions among HR-HPV positive are needed and this will improve cervical cancer screening and reduce the cost associated with patient care. As discussed below, recent advancements in proteomic research have yielded techniques to aid in novel protein biomarker identification.

Because the cause of most human diseases lies in the functional dysregulation of protein interactions, advances in proteomic technologies represent exciting ways to explore the disease processes at the molecular level. The identification, quantification, classification and functional assignment of proteins are essential to the full understanding of these molecular events. Since mammalian systems are much more complex than can be deciphered by their genes alone, expression analysis directly at the protein level is thought to be necessary to unravel the critical changes that occur as part of pathogenesis of a disease (Srinivas et al. 2001).

The surface-enhanced laser desorption/ionization (SELDI) time of flight (TOF) mass spectrometry (MS) is one such technique with considerable potential for the detection and quantitation of protein in a wide variety of human tissue samples (Wright et al. 1999) and appear to be an important diagnostic tool for a whole range of diseases. Preliminary applications of proteomic techniques are published in the area of cervical cancer using two-dimensional gel analysis (Bae et al. 2005) and SELDI (Wong et al. 2004). These studies demonstrate the ability of proteomics approach to distinguish cervical cancer from its normal counterpart. If this approach is also workable in the analysis of non invasive samples such as plasma, urine, exfoliated cervical cells or cervical mucous, it might potentially be used in the diagnosis of underlying precancerous cervical lesions in high-risk women. To our knowledge, there are no published reports demonstrating the usefulness of SELDI protein profiles to identify HR-HPV positive subjects who are likely to have underlying true precancerous lesions of the cervix.

The current study is an exploratory study to identify candidate SELDI-TOF MS protein profiles in plasma that may aid in distinguishing CIN 3 from CIN 1 among women positive for HR-HPV.

## Materials and Methods

### Samples

The plasma samples used were from a previously conducted study of cervical neoplasia (nutrition ancillary study of the ASCUS/LSIL Triage Study for cervical cancer (ALTS) where HPV status (as detected by the Hybrid Capture 2 assay-Digene Diagnostics) and cervical diagnoses were known (Piyathilake et al. 2004). Plasma samples from sixty HR-HPV positive women diagnosed with CIN 1 (n = 28) and CIN 3 (n = 32) were randomly selected from the prior study and were used in this study. A randomized template that was generated by the statistician and that included triplicate samples from each case (CIN 3) and control (CIN 1) plasma sample was developed to ensure samples were run randomly on the system. Case-control status was kept blinded and the protein profile data generated as described below was sent directly to the statistician for analysis.

### Methods

The plasma samples were prepared in triplicate and diluted 1:10 in binding buffer (100mM Ammonium Acetate pH 4.5, 0.1% Triton X-100) using the Biomek 2000 Robotics System (Beckman Coulter). The plasma samples (100 μl) were added to a pre-washed bioprocessor containing the WCX2, eight spot protein chips (Ciphergen Biosystem, Inc). The samples were incubated on a shaker for 2 hours at room temperature. The chips were washed twice with binding buffer and twice with HPLC grade H_2_0. The bioprocessor was disassembled and the chips were washed several times with HPLC grade H_2_0. Each spot was allowed to air dry and 0.5 μl of sinapinic acid (energy absorbing molecule) was added twice to each spot. The chips were read on the Ciphergen Protein Chip Reader Series PBS II using the following parameters: Molecular Weight range–1000 to 15,000, High Mass–20,000, Intensity–180, and Sensitivity–7.

## Statistical Analysis

### Pre-processing of data and creating training and test sets

The mean spectrum for an individual was obtained by averaging across the three replicate spectra for that individual. The m-z values for the CIN 3 group were slightly different from the CIN 1 group, but these differences were within the expected margin of error (0.2%) of the instrument. The average m-z values of both groups at each point were used as the estimated m-z value. The error of +/– 0.2% was used to develop a new variable index, which was the upper limit of the m-z interval within this error range. A total of 530 index variables were used to represent the 10,300 m-z values. Peaks were defined as the maximum intensity value within each set of m-z values represented by the new variable index. Each group had 530 peaks. The data sets were then randomly divided into training and test sets. Six patients from each group were randomly selected to create the test set. Therefore, the training set consisted of 28 CIN 3 and 22 CIN 1.

### Principal component analysis (PCA)

PCA plot which places the components in terms of the proportion of variance in the data that is attributable to the particular combination was used to visually demonstrate the differences in the groups before building a classifier in order to assess the possibility of obtaining a good classifier. The PCA components are orthogonal to each other. Therefore, the first principal component accounts for the largest amount of variability and the second component accounts for the largest amount of variability unaccounted for by the first. The orthogonality of the components allows their representation as axes in a two-dimensional plot. If the variability explained by the components is the variability associated with the differences in the two groups (CIN 3 and CIN 1), and not the random noise, the principal component plot will visually demonstrate the differences between the groups.

### Building a classifier

An iterative process was implemented to develop a classifier. At each iteration, a random sample of 18 CIN 1 and 22 CIN 3 was selected from the training data set. The peaks that were statistically significantly different based on a Wilcoxon Rank Sum test were then subjected to a stepwise discriminant analysis. This procedure produced a list of index values at which the intensities were most likely to separate the two groups. This list of possible discriminators was stored. The entire process was repeated 300 times in this preliminary analysis. Index values that occurred in at least 270 of the 300 (90%) lists were selected to put into the classifier. This error rate of the classifier was estimated by using the classifier to classify the groups in the test data set.

## Results

**Figure 1.**
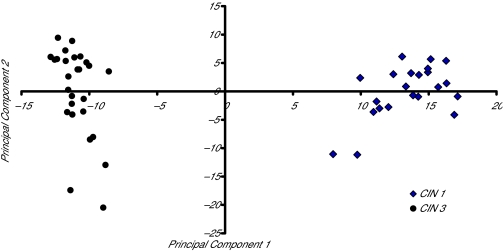
PCA plot between CIN 3 and CIN 1.

**Figure 2.**
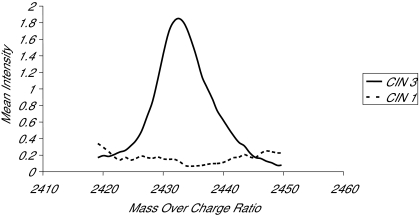
The region of the spectra of CIN 3 and CIN 1 groups that provided 100% separation between the groups.

The PCA was based on the entire set of 530 index values. The first two components explained 37 percent of the variability in the data. As shown in Figure 1, the PCA plot between CIN 3 and CIN using these first two components suggested that it is possible to obtain a good separation between the groups. There were five regions in the spectra that occurred in 90% of the lists produced by the stepwise discriminant procedure. These consisted of the region between m_z 2261–2282b (index 28, 29), m_z 2371–2392 (index, 38), and m_z 2426–2437 (index 43, 44). As shown in Figure 2, a subset of these five m_z regions i.e. m_z 2426–2437 groups provided 100% separation between the groups.

## Discussion

An important application of SELDI is the simultaneous analysis of multiple proteins to establish “fingerprint” profiles that discriminate disease from non-disease. This is an important approach since no single biomarker or protein by itself will improve the early detection/diagnosis of diseases including cancer or pre-cancer. Protein based approaches are utilized to study the natural history and treatment of several cancers including ovarian (Lin et al. 2006), breast (Wulfkuhle et al. 2001), colon/colorectal (Lawrie et al. 2001; Ward et al. 2006; Engwegen et al. 2006) prostate (Waghray et al. 2001), head and neck (Roesch-Ely et al. 2006), endometrial (Zhu et al. 2006), nasopharynx (Ho et al. 2006), bladder (Langbein et al. 2006), hepatocellular (Ward et al. 2006) and gastric cancer (Liang et al. 2006). Protein profiling of body fluids such as nipple aspirate fluid (NAF) has shown to be rapid, reproducible, and capable of identifying protein signatures that appear to differentiate NAF samples from breast cancer patients and healthy controls, including those with an abnormal mammogram who were later proven to be biopsy normal (Paweletz et al. 2001). The use of SELDI protein profiles to detect putative breast cancer markers in peripheral samples such as saliva has also been reported in recent studies (Streckfus et al. 2006). A few studies have reported on the ability of serum protein profiles to separate benign hyperplasia from cancer and non-cancer tissues (Qu et al. 2002). Although in virology, much more limited use has been made of proteomics, use of several proteomic approaches for the analysis of HIV-cellular receptor interactions, the molecular mechanisms of transport of herpes simplex virus within neurons, and the structure of the tegument of herpes simplex virus is beginning to be appreciated (Bernhard et al. 2005).

Our preliminary study demonstrated that plasma protein profiles are significantly different between women diagnosed with CIN 1 and CIN 3. Biologically, it is plausible to see difference in protein profiles between HPV positive women who develop CIN 3 and HPV positive women who don’t develop CIN 3 because several biological pathways related to HPV (example: HPV integration which may result in development of CIN 3 lesions) could be different between HPV positive women who develop CIN 3 and who don’t. Cervical epithelial cells exposed to HPV which undergo transformation to pre-neoplastic lesions such as CIN 3 are very likely to differentially express signaling molecules responsible for host responses following HPV infection that resulted in CIN 3 and these molecules are likely to be present in the microenvironment of cervical cells and in the circulation. Identification of such molecules will have enormous clinical applications. Triage of HPV positive women by protein biomarkers could yield enormous cost savings and other important benefits include less invasive sample collection and inconvenience regarding referral.

These results, however, have to be considered with caution. SELDI-TOF-MS analysis is a very sensitive method for determining differences in protein spectra of complex samples including plasma and serum. Because of this sensitivity, the method is very susceptible to the effects of various biases. Since cases of CIN 3 and of CIN 1 were selected randomly from participants from the ALTS Nutrition Ancillary Study, we know of no biases which separate the samples of CIN 3 from those of CIN 1. Similarly, because the triplicate aliquots of the samples were run randomly, there should be no bias in the analysis. Before final conclusions can be drawn, however, it is necessary to further evaluate this classification model by using an independent replication set, which will allow more precise conclusions about the accuracy of this classification. These pilot results also warrant an expanded study using SELDI-TOF-MS analysis to evaluate samples of plasma and other samples (cervical cells and cervical mucous) from patients with a wide range of cervical lesions to determine if these can be classified successfully. A study is currently underway at the University of Alabama (U54 CA118948) to generate this data.
